# Root Differentiation of Agricultural Plant Cultivars and Proveniences Using FTIR Spectroscopy

**DOI:** 10.3389/fpls.2018.00748

**Published:** 2018-06-05

**Authors:** Nicole Legner, Catharina Meinen, Rolf Rauber

**Affiliations:** Division of Agronomy, Department of Crop Sciences, University of Goettingen, Goettingen, Germany

**Keywords:** *Avena sativa*, *Pisum sativum*, *Zea mays*, *Echinochloa crus-galli*, cluster analysis, root differentiation, species differentiation, FTIR-ATR spectroscopy

## Abstract

The differentiation of roots of agricultural species is desired for a deeper understanding of the belowground root interaction which helps to understand the complex interaction in intercropping and crop-weed systems. The roots can be reliably differentiated via Fourier transform infrared spectroscopy with attenuated total reflection (FTIR-ATR). In two replicated greenhouse experiments, six pea cultivars, five oat cultivars as well as seven maize cultivars and five barnyard grass proveniences (n = 10 plants/cultivar or provenience) were grown under controlled conditions. One root of each plant was harvested and five different root segments of each root were separated, dried and measured with FTIR-ATR spectroscopy. The results showed that, firstly, the root spectra of single pea and single oat cultivars as well as single maize and single barnyard grass cultivars/proveniences separated species-specific in cluster analyses. In the majority of cases the species separation was correct, but in a few cases, the spectra of the root tips had to be omitted to ensure the precise separation between the species. Therefore, species differentiation is possible regardless of the cultivar or provenience. Consequently, all tested cultivars of pea and oat spectra were analyzed together and separated within a cluster analysis according to their affiliated species. The same result was found in a cluster analysis with maize and barnyard grass spectra. Secondly, a cluster analysis with all species (pea, oat, maize and barnyard grass) was performed. The species split up species-specific and formed a dicotyledonous pea cluster and a monocotyledonous cluster subdivided in oat, maize and barnyard grass subclusters. Thirdly, cultivar or provenience differentiations within one species were possible in one of the two replicated experiments. But these separations were less resilient.

## Introduction

The use of infrared (IR) spectroscopy for biological samples started in the 1950s (Barer et al., 1949), and was soon also applied for the identification of microorganisms (Thomas and Greenstreet, 1954). IR spectroscopy is recording the spectral pattern of a sample and representing the chemical composition as a function of wavenumber between 400 and 4,000 cm^−1^ (Griffiths and de Hasetz, 2007; Chalmers and Griffiths, 2017). After remarkable adjustments and improvements of the IR spectroscopy, the Fourier transform infrared (FTIR) spectroscopy became a powerful tool for the differentiation and identification of different samples. Not only gases, liquids and solids could be analyzed, but also the investigation of biological samples improved tremendously. The spectra could now be measured significantly faster (from 30 min to within a minute for each sample) and with higher sensitivity (Griffiths and de Hasetz, 2007). The chemical composition of the sample can be analyzed as simple molecules show the specific absorption bands in the FTIR spectra. Whereas, complex biological samples show broader and/or diverse absorption peaks in the FTIR spectra (Griffiths and de Hasetz, 2007). This results from different chemical bonds which can overlap. Certain wavenumber ranges can be assigned to key compounds of the sample, as for example, proteins to the range 1,800 to 1,485 cm^−1^ (Naumann, 2009; Naumann et al., 2010). The research of biological samples with FTIR spectroscopy started again with bacteria (Naumann et al., 1988a,b). For identifying microorganisms, strains or isolates, different techniques of FTIR spectroscopy can be applied. These techniques are transmission, which was most commonly used, as well as microspectroscopy, spectral or diffuse reflectance. The attenuated total reflection (ATR) scanning technique was used more and more (Filip and Hermann, 2001; Mariey et al., 2001; Filip et al., 2004). The FTIR-ATR spectroscopy became an appropriate method for biological samples due to the fact that the samples preparation is minimized because the ATR measurement is independent of samples thickness (Kazarian and Chan, 2006) and requires only small amounts of sample material. As sampling material also fresh material can be used which spares sampling preparation time. Additionally, the short measuring times provide the possibility of high-throughput screening (Cozzolino, 2014; Meinen and Rauber, 2015). Therefore, FTIR-ATR spectroscopy is a very reliable method which is easy in handling with fast data acquisition.

The roots of plant species can be differentiated by FTIR spectroscopy. It was demonstrated that distantly related species as pea (*Pisum sativum* L.) and oat (*Avena sativa* L.) are distinguishable from each other (Naumann et al., 2010), as well as closer related species as e.g., maize (*Zea mays* L.) and barnyard grass (*Echinochloa crus-galli* (L.) P. Beauv.) or wheat (*Triticum aestivum* L.) and blackgrass (*Alopecurus myosuroides* Huds.) (Rewald et al., 2012; Meinen and Rauber, 2015). The differentiation of roots of different species is crucial for getting a deeper understanding of the belowground root interaction. This interaction can occur between different plants of one species (intraspecific) or between plants of different species (interspecific). Intraspecific and interspecific interaction can be found in the field, e.g., in intercropping systems (Li et al., 2006), between crops and weed species (Ehrmann and Ritz, 2014) and in pots under controlled conditions (Hauggaard-Nielsen and Jensen, 2005). Intercropping often includes certain advantages, e.g., complementary resource use or yield increase (Helenius and Jokinen, 1994; Hauggaard-Nielsen et al., 2006) and was frequently investigated with the focus of above-ground interactions (e.g., Rauber et al., 2001; Lauk and Lauk, 2008; Dordas et al., 2012). The belowground reaction of the species is more difficult to assess but the knowledge about the root distribution patterns may help to explain a part of the belowground interactions. Weed species, e.g., barnyard grass, interact with crop species and are able to be strong resource competitors, above- and belowground, reducing the yield of the crops (Travlos et al., 2011). How the root distribution patterns of species influence each other is difficult to access and is therefore of great interest to investigate. As the visual differentiation between the roots of different species is not always possible or not absolutely reliable (Rewald et al., 2012), the analysis by FTIR spectroscopy has the potential to close this gap.

In the FTIR studies of higher plants, only one cultivar or provenience per species is commonly used (Naumann et al., 2010; Meinen and Rauber, 2015). In contrast, studies of bacteria used strains or different isolates and proved these to be specifiable (e.g., Mariey et al., 2001). Accordingly, different cultivars or proveniences of higher plant species should be investigated to prove that the differentiation of species is not dependent of the cultivar or proveniences. To prove species differentiability independently of provenience or cultivar by FTIR spectroscopy, the present work used different cultivars of the species pea and oat as distantly related species as well as different cultivars of maize and different proveniences of barnyard grass as closer related species in greenhouse experiments.

The aim of the present work was to study the spectral intra- and interspecific variability between species and between their cultivars/proveniences. We hypothesized that root spectra of (i) each cultivar of pea is distinguishable from each cultivar of oat as well as each cultivar of maize from each provenience of barnyard grass (1:1 comparison). Accordingly, the species pea and oat as well as the species maize and barnyard grass can be differentiated from each other when all cultivars/proveniences are used. We further hypothesized that (ii) the species pea, oat, maize and barnyard grass could be separated when using all cultivars/proveniences in one cluster analysis and (iii) the cultivar/provenience differentiation within a species is possible.

## Materials and methods

### Plant material and experiments

**Figure 1:**
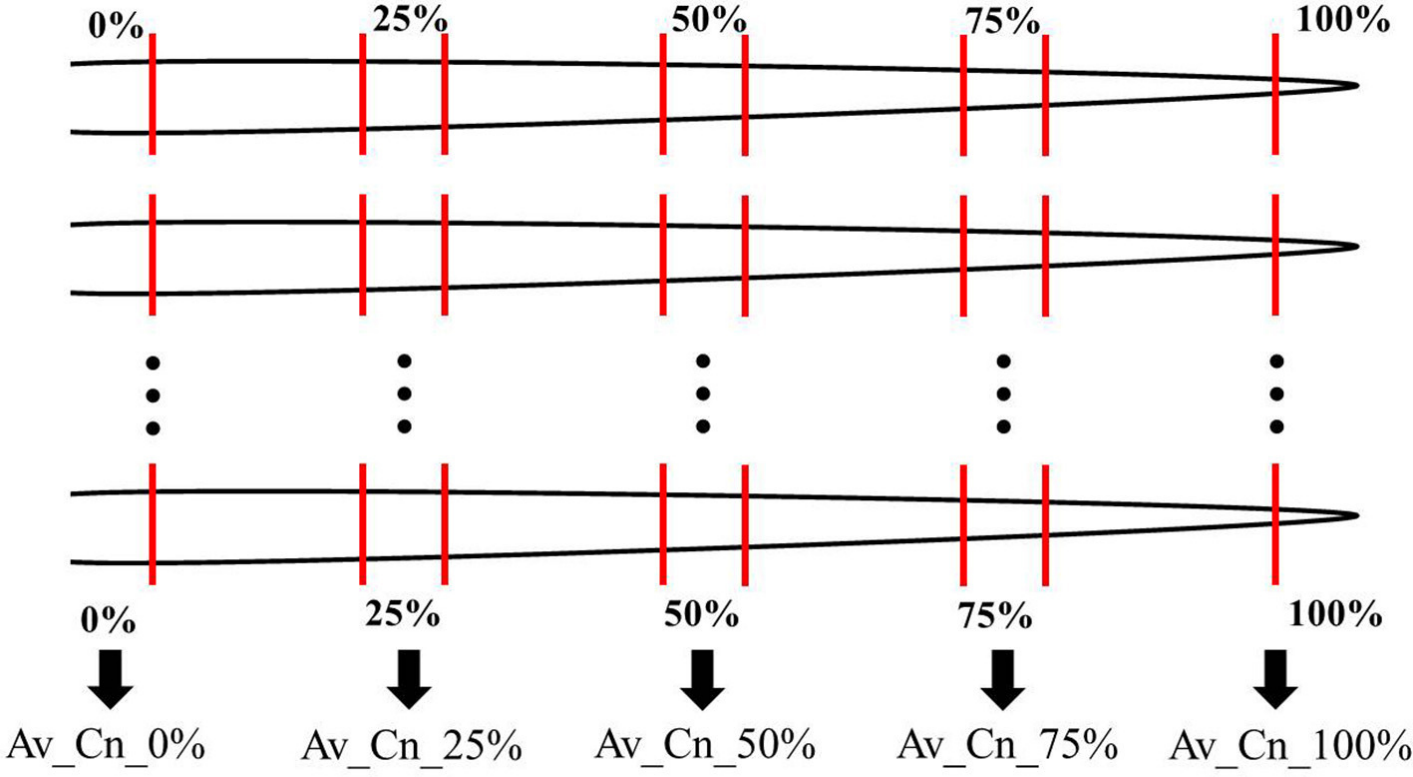
Root sampling scheme for all experiments. From every root, five root segments of 1 cm length were taken at the root basis (0%), the root tip (100%), the middle of the root (50%) and further two in between (25 and 75%). The FTIR-ATR spectra of the corresponding root position of one cultivar or provenience was used to calculate an arithmetic average (Av) which are labeled “Av_Cn_0%” in the figures. In the text, the abbreviation “Av-spectra” is used. Cn means cultivar number. See Table 2 for the number of roots used for the averages.

In two greenhouse experiments in 2013 and 2014, six cultivars of the species pea (*Pisum sativum* L.) and five cultivars of the species oat (*Avena sativa* L.) as distantly related species as well as seven cultivars of maize (*Zea mays* L.) and five different proveniences of the species barnyard grass [*Echinochloa crus-galli* (L.) P. Beauv.] as closely related species were investigated (Table 1). The experiment with pea and oat cultivars took place on October 14 to November 4, 2013 (exp. 1-1) and was repeated on February 17 to March 10, 2014 (exp. 1-2). The experiment with maize cultivars and barnyard grass proveniences was conducted on October 28 to November 11, 2013 (exp. 2-1) and was repeated on February 24 to March 17, 2014 (exp. 2-2). The cultivars and proveniences (n = 10 plants for each cultivar and provenience) grew individually in 1 l pots with 11 cm diameter in a soil-sand mixture (1:1, pH 7.6) in the greenhouse. The soil came from the experimental farm Reinshof (south of Goettingen, silty loam) and was sterilized for 12 h at 100°C. The pots were regularly irrigated and no further fertilizer was used. The pots with barnyard grass seeds were stratified for 7 days at 5°C in a cooling chamber. For each experiment, the pots were arranged in a randomized plot design and surrounded with an additional row of plants of the involved species to prevent edge effects. Additional lamps (HPS 400 W, Co. Hortilux Schreder with Son-T Agro 400 W, Co. Philips, medium reflector) provided constant light ~300 μmol m^−2^ s^−1^ from 6 a.m. to 10 p.m. The air temperature was measured constantly and remained at ~24°C in the daytime and at ~17°C at night-time for all experiments. After 21 days, the plants growth stages were recorded according to the BBCH-scale for cereals, maize and pea respectively (Meier, 1997). At the end of the experiments 1-1 and 1-2, pea developed six tendrils (BBCH stage 16) and oat showed three unfolded leaves (BBCH stage 13 in exp. 1-1) or accordingly developed two tillers (BBCH stage 22 in exp. 1-2). At the end of the experiment 2-1, maize showed four unfolded leaves (BBCH stage 14) and barnyard grass three unfolded leaves (BBCH stage 13). At the end of the experiment 2-2, maize showed five leaves unfolded (BBCH stage 15) and in barnyard grass the first tiller was detectable (BBCH stage 21). The thickest and longest root was harvested which was either the seminal or the first lateral root when the seminal root was broken or nonexistent; sometimes even the thickest adventitious root was harvested. In most cases only one root per plant and pot was harvest but for oat of the experiment 1-1 two roots were harvested and used for the evaluation: one long and thin one and one thick root. There were only minimal differences in the thin root spectra compared to the thick root spectra. Furthermore, a differentiation in a cluster analysis was not possible between the thin and the thick roots. Hence both spectra were included in the further analysis. This had also the advantage of increasing the sample size. From each harvested root of all experiments, five root segments of 1 cm length were sampled: root basis (0%), middle of the root (50%), root tip (100%) and further two in between (25 and 75%; Figure 1). All root segments were dried at 60°C for 48 h.

**Table 1:** Cultivars and proveniences of the different species used for the experiments.

**Pea -** ***Pisum sativum*** **ssp**. ***sativum***			**Oat -** ***Avena sativa***		
Ps1	convar. *sativum*-leafed	Bohatyr	As1	White oat	Ivory
Ps2	convar. *sativum*-semi-leafless	Respect	As2	Yellow oat	Flämingsgold
Ps3	convar. *arvense*-winter pea	EFB 33	As3	Black oat	Zorro
Ps4	convar. *medullare*-wrinkled pea	Salzmünder Edelperle	As4	Hulless oat	Samuel
Ps5	convar. *arvense*-field pea	Lisa	As5	Winter oat	Fleuron
Ps6	convar. *axiphium*-sugar pea	Zuckearfen			
**Maize -** ***Zea mays***			**Barnyard grass -** ***Echinochloa crus-galli***		
Zm1	var. *indurata*-flint maize	Gelber Badischer Land	Ec1	Germany	Göttingen, GH 2009
Zm2	var. *everta*- popcorn maize	Pink Pop	Ec2	Greece	Veria 2013
Zm3	var. *indentata*-dent maize	KXA 0221	Ec3	Canada	GH 2009
Zm4	var. *saccharata*-sweet corn	Golden Bantam	Ec4	China	Manchuria 2013
Zm5	early ripening, K160	Lorado	Ec5	Poland	Mydlniki 2009
Zm6	var. *tunicata*-black pod corn	Plume Divinite Vetu			
Zm7	late ripening, K290	Surreal			

*Given are the cultivar names and the provenience origins. GH stands for greenhouse. K stands for the maturity number of the grain maize*.

### FTIR measurements and evaluation

Every dried root segment was measured in the middle of the segment with the FTIR-ATR spectrometer (Alpha-P, Bruker Optics, Ettlingen, Germany) with a resolution of 4 cm^−1^ and 24 scans in the spectral range of 3,997–374 cm^−1^. The FTIR-ATR spectra were evaluated with the software OPUS (version 7.2, Bruker Optics, Ettlingen, Germany). An arithmetic average (Av) of the FTIR spectra was calculated for each root position (0, 25, 50, 75, and 100%) (see Figure 1) for each cultivar/provenience (see Table 1) including different replications (Table 2). Referring to these spectral averages, the term “Av-spectra” will be used in the following text. To illustrate graphically the averaging of the measured FTIR spectra, each 10 FTIR spectra of the middle root segment of pea (Supplementary Figure 1A, 50%), oat (Supplementary Figure 1B), maize (Supplementary Figure 1C), and barnyard grass (Supplementary Figure 1D) were displayed in blue and the corresponding Av-spectra of these segments in red. Additionally to the evaluation of each experiment on its own, the experiments 1-1 and 1-2 as well as 2-1 and 2-2 were united and subsequently evaluated together. Referring to these results, the term “united experiment 1” as well as “united experiment 2” was used (Table 2). With the Av-spectra, hierarchical cluster analyses were calculated.

**Table 2:** Sample size for each five Av-spectra of the different species, cultivars, provinces, and experiments. Ec2 in exp. 2-1 did not germinate. For each cultivar/provenience10 plants grew individually in pots. From each plant, one root was extracted to gain the five root segments. Over each root position the Av-spectra was averaged (see Figure 1). The exception was experiment 1-1, in several cases two roots were harvested which led to a higher sample size for the Av-spectra.

	**exp. 1-1**	**exp. 1-2**	**united exp. 1^a^**		**exp. 2-1**	**exp. 2-2**	**united exp. 2^b^**
Ps1	11–14	10	21–24	Zm1	10	10	20
Ps2	12–13	9–10	21–23	Zm2	10	10	20
Ps3	11–14	8–10	21–24	Zm3	10	7–10	17–20
Ps4	11–12	10	21–21	Zm4	10	10	20
Ps5	10–12	9–10	19–22	Zm5	10	9–10	19–20
Ps6	11–12	10	21–22	Zm6	10	10	20
				Zm7	10	10	20
As1	21	10	31				
As2	23	10	33	Ec1	10	9–10	19–20
As3	19–20	10	29–30	Ec2	-	7–10	7–10
As4	20	10	30	Ec3	10–11	7–11	18–21
As5	20–21	9–10	30–31	Ec4	9–10	9–10	19–20
				Ec5	10	8–10	18–20

a *the experiments 1-1 and 1-2 were united and subsequently evaluated together*.

b *the experiments 2-1 and 2-2 were united and subsequently evaluated together*.

For the cluster analysis, a reduced spectral range (3,751–2,749 cm^−1^ and 1,801–599 cm^−1^) as well as the complete spectral range (3,997–374 cm^−1^) was utilized. It was tested whether the complete or a reduced frequency range achieved better results at the calculation of the cluster analysis. In almost all cases both frequency ranges came to similar results though in most cases the reduced frequency range showed better results by reaching higher heterogeneity without affecting the general outcome. This is in accordance to Meinen and Rauber (2015), Naumann (2009), and Naumann et al. (2010) who also used a reduced frequency range.

For the data processing, different standard evaluations of the software OPUS were tested. The first derivation (with nine smoothing points) and vector normalization as well as the second derivation (with nine smoothing points) and vector normalization was used, both followed by Ward's algorithm as well as Euclidian distance. Ward's algorithm attempts to find homogeneous groups. The two homogeneous groups which possess the least increase of heterogeneity are merged into a new cluster. To distinguish between species the first derivation/vector normalization was most suitable (which was already shown in Naumann et al., 2010; Meinen and Rauber, 2015), but to distinguish between the cultivars/proveniences within one species, only the second derivation/vector normalization achieved satisfying results. In every figure, it is marked clearly which data processing was executed.

Firstly, 1:1 comparisons were conducted. A 1:1 comparison is a cluster analysis of the Av-spectra of one single cultivar/provenience of one species with the Av-spectra of another single cultivar/provenience of a second species. For the data processing, the first derivation followed by vector normalization, Ward's algorithm and Euclidian distance of the reduced spectral range was used. Here, the differences between the species could be elaborated regardless of the cultivar or provenience. Subsequently, the Av-spectra of all cultivars/provenience of one species were used with the Av-spectra of all cultivars/proveniences of another species in cluster analyses to assure the species differentiation when all cultivars/proveniences are included. Therefore, the first derivation followed by vector normalization, Ward's algorithm and Euclidian distance of the reduced spectral range was used. These analyses showed results with a higher heterogeneity than the 1:1 comparisons because the higher number of Av-spectra gave a greater variance and therefore stabilized the results. Secondly, the Av-spectra of all species/proveniences were used in one cluster analysis. Again, the first derivation followed by vector normalization, Ward's algorithm and Euclidian distance of the reduced spectral range was used. Here, the differentiation of the species and the kinship between the species were investigated. And thirdly, the Av-spectra of all cultivars/proveniences of one species were used for cluster analyses to evaluate if a separation of the cultivars/proveniences within one species is possible. For the species pea, oat and barnyard grass, the second derivation followed by vector normalization, Ward's algorithm and Euclidian distance of the complete spectral range was applied. For maize, the second derivation followed by vector normalization, Ward's algorithm and Euclidian distance of the reduced spectral range were used.

## Results

### Species differentiation

**Figure 2:**
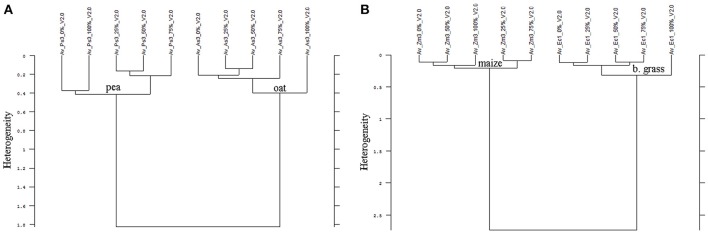
Cluster analysis of the Av-spectra of the 1:1 comparision of **(A)** *Pisum sativum* (Ps, pea) and *Avena sativa* (As, oat) as well as **(B)** *Zea mays* (Zm, maize) and Echinochloa crus-galli (Ec, barnyard grass). Here exemplarily represented Ps3 and As3 of experiment 1-2 (A) as well as Zm3 and Ec1 of experiment 2-2. Entire results are shown in Supplementary Tables 1, 2. Cluster analysis was evaluated with the first derivation and vector normalization of the reduced frequency range (3,751–2,749 cm^−1^ and 1,801–599 cm^−1^), Ward's algorithm and Euclidian distance. The Av-spectra are averages (Av) of FTIR-ATR spectra of the different root segments (100% root tip, 0% root basis etc.; see Figure 1 for scheme, Table 2 for n, Table 1 for names of cultivars and proveniences).

**Figure 3:**
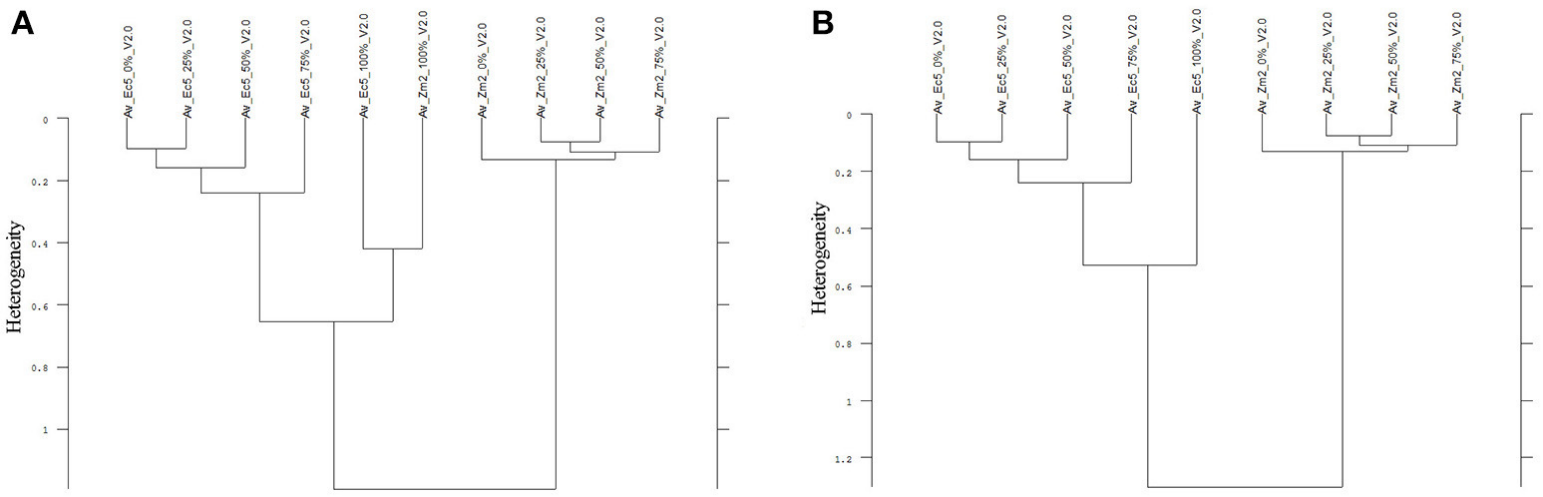
Cluster analysis of the Av-spectra of the cultivar 2 of *Zea mays* (Zm2, maize) and the cultivar 5 of *Echinochloa crus-galli* (Ec5, barnyard grass) for the experiment 2-2 with all 10 Av-spectra **(A)** and with the omission of the 100% Av-spectra (root tip) of barnyard grass (Av_Ec5_100%) **(B)**. Cluster analysis was evaluated with the first derivation and vector normalization of the reduced frequency range (3,751–2,749 cm^−1^ and 1,801–599 cm^−1^), Ward's algorithm and Euclidian distance. The Av-spectra are averages (Av) of FTIR-ATR spectra of the different root segments (100% root tip, 0% root basis etc.; see Figure 1 for scheme, Table 2 for n, Table 1 for names of cultivars and proveniences).

**Figure 4:**
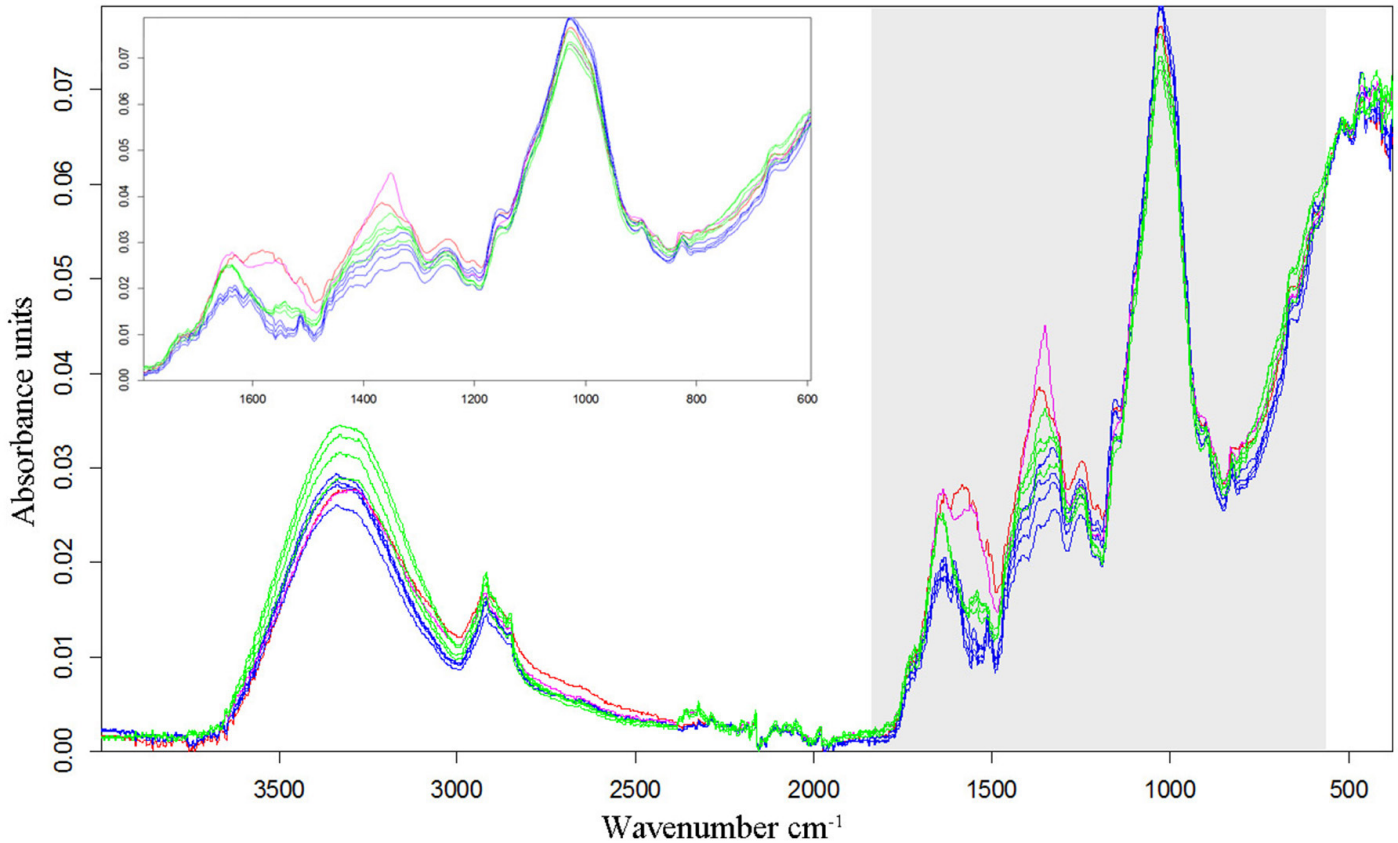
Av-spectra of the maize cultivar 2 (Zm2) and the barnyard grass cultivar 5 (Ec5). The four maize Av-spectra of the root segments 0–75% are displayed in blue, the maize Av-spectra of the root segment 100% (root tip) is display in red. The four barnyard grass Av-spectra of the root segments 0–75% are displayed in green, the barnyard grass Av-spectra of the root segment 100% (root tip) is display in pink. Spectra are vector-normalized and offset-corrected. As detail the Av-spectra of the wavenumber 1,800–600 cm^−1^ are illustrated.

In all experiments, the species differentiation on the basis of the different root segments was successfully conducted. Firstly, comparing the root segments of each cultivar of pea with each cultivar of oat (1:1 comparison, cluster analysis with reduced frequency range and first derivation data evaluation), experiment 1-1 as well as the united evaluation of the experiment 1 could always distinguish clearly between pea and oat (Figure 2A as example, detailed results in the Supplementary Table 1). In experiment 1-2, most of the 1:1 comparisons of oat cultivar 2 (As2) and cultivar 4 (As4) with each pea cultivar were not able to separate between pea and oat. The non-existing separation was caused by the 100% Av-spectra (root tip). Leaving these 100% Av-spectra out of the cluster analysis (marked with an asterisk in the Supplementary Table 1), the roots segments of each pea cultivar could be clearly separated from each oat cultivar. The 1:1 comparison of each cultivar of maize with each provenience of barnyard grass (cluster analysis with reduced frequency range and first derivation data evaluation), the united experiment 2 (exp. 2-1 and 2-2) was able to separate clearly between the root segments of maize cultivars and of barnyard grass proveniences (Figure 2B as example, detailed results in the Supplementary Table 2). The cluster analyses of experiment 2-2 separated between the two species in all cases but one: the maize cultivar 2 (Zm2) could not be separated from the barnyard grass provenience 5 (Ec5) (Figure 3A). Again, leaving the 100 %-spectra out of the cluster analysis, the separation was possible (marked with an asterisk in the Supplementary Table 2) (Figure 3B). To illustrate this difficulty, the Av-spectra of the two cultivars were display in different colors: The Av-spectra of the root segments 0–75% of maize (Zm2) were displayed in blue whereas the Av-spectra of the root segment 100% of maize (Zm2) in red (Figure 4). The Av-spectra of the barnyard grass cultivar five were displayed in green (0–75%, Ec5) and in pink (100%, Ec5). In the detail of Figure 4, it is clearly visible that the red line (Av_Zm2_100%) is not in the striking distance of the blue lines (Av_Zm2_0–75%). It is much closer to the pink line (Av_Ec5_100%). The pink line has also a little distance from their corresponding green lines (Av_Ec5_0–75%) but it is much closer to the associated green lines. Hence, with the omission of the root segment 100% of maize (Av_Zm2_100%) it was possible to separate in a cluster analysis between Zm2 and Ec5 (Figure 3B). In experiment 2-1, it was not possible to separate between the roots segments of maize and barnyard grass in several cases. The 100% Av-spectra caused the non-separations and leaving these spectra out of the cluster analysis, the separation was possible in all cases (see Supplementary Table 2 for details).

**Figure 5:**
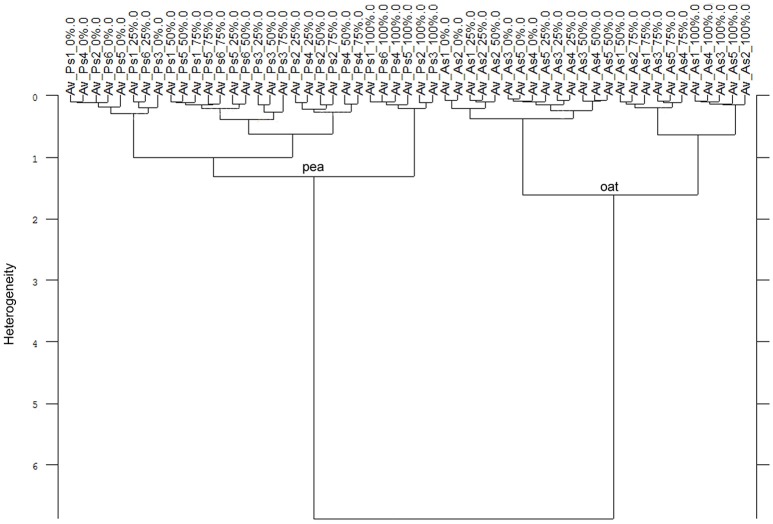
Cluster analysis of the Av-spectra of all cultivars of the species *Pisum sativum* (Ps, pea) and *Avena sativa* (As, oat) for the united experiment 1 (exp. 1-1 and 1-2). Cluster analysis was evaluated with the first derivation and vector normalization of the reduced frequency range (3,751–2,749 cm^−1^ and 1,801–599 cm^−1^), Ward's algorithm and Euclidian distance. The Av-spectra are averages (Av) of FTIR-ATR spectra of the different root segments (100% root tip, 0% root basis etc.; see Figure 1 for scheme, Table 2 for n, Table 1 for names of cultivars and proveniences).

**Figure 6:**
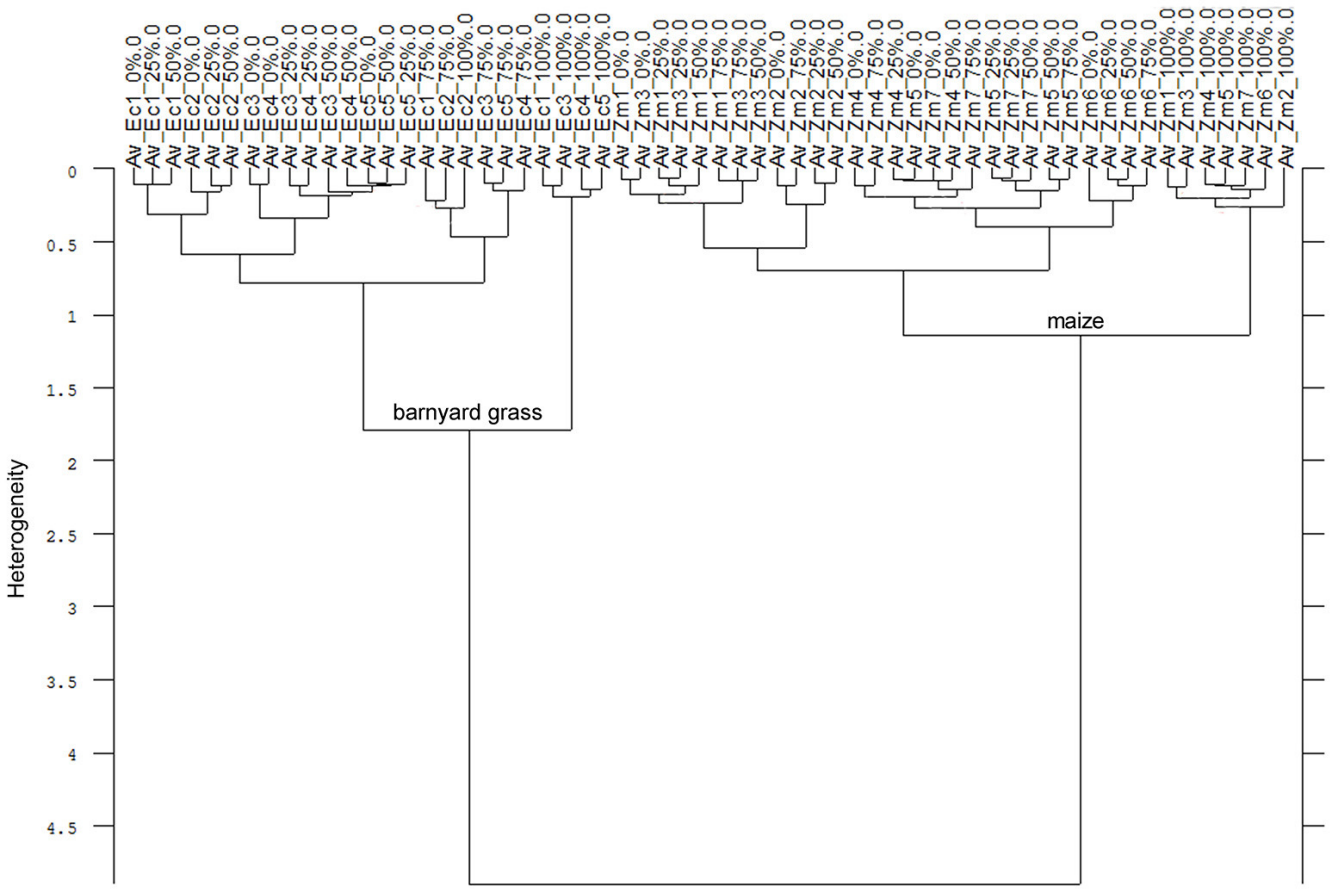
Cluster analysis of the Av-spectra of all cultivars of the species *Zea mays* (Zm, maize) and all proveniences of the species *Echinochloa crus-galli* (Ec, barnyard grass) for the united experiment 2 (exp. 2-1 and 2-2). Cluster analysis was evaluated with the first derivation and vector normalization of the reduced frequency range (3,751–2,749 cm^−1^ and 1,801–599 cm^−1^), Ward's algorithm and Euclidian distance. The Av-spectra are averages (Av) of FTIR-ATR spectra of the different root segments (100% root tip, 0% root basis etc.; see Figure 1 for scheme, Table 2 for n, Table 1 for names of cultivars and proveniences).

Consequently, the Av-spectra of the roots of the far related species pea and oat were clearly separated in the cluster analysis of the united experiment 1 (exp. 1-1 and 1-2, Figure 5). Here the interspecific heterogeneity was again high (6.88), whereas the intraspecific one was low in pea (1.32) and oat (1.60). Similar results with a clear separation of pea and oat root segments were obtained when conducting the cluster analysis separately for experiment 1-1 and for the experiment 1-2. The Av-spectra of the root segments of the close related species maize and barnyard grass of the united experiment 2 (exp. 2-1 and 2-2) were precisely separated from each other by cluster analysis (Figure 6). The interspecific heterogeneity was 4.90 and the intraspecific was 1.14 (maize) and 1.78 (barnyard grass). This result could also be achieved for the separate evaluation of the experiments 2-1 and 2-2. Here, it was not necessary to leave out any 100% Av-spectra in the cluster analysis. Due to the fact that a higher amount of Av-spectra were included in the cluster analysis, the species separation was achieved even if some of the 100% Av-spectra were more heterogeneous compared to the other Av-spectra.

**Figure 7:**
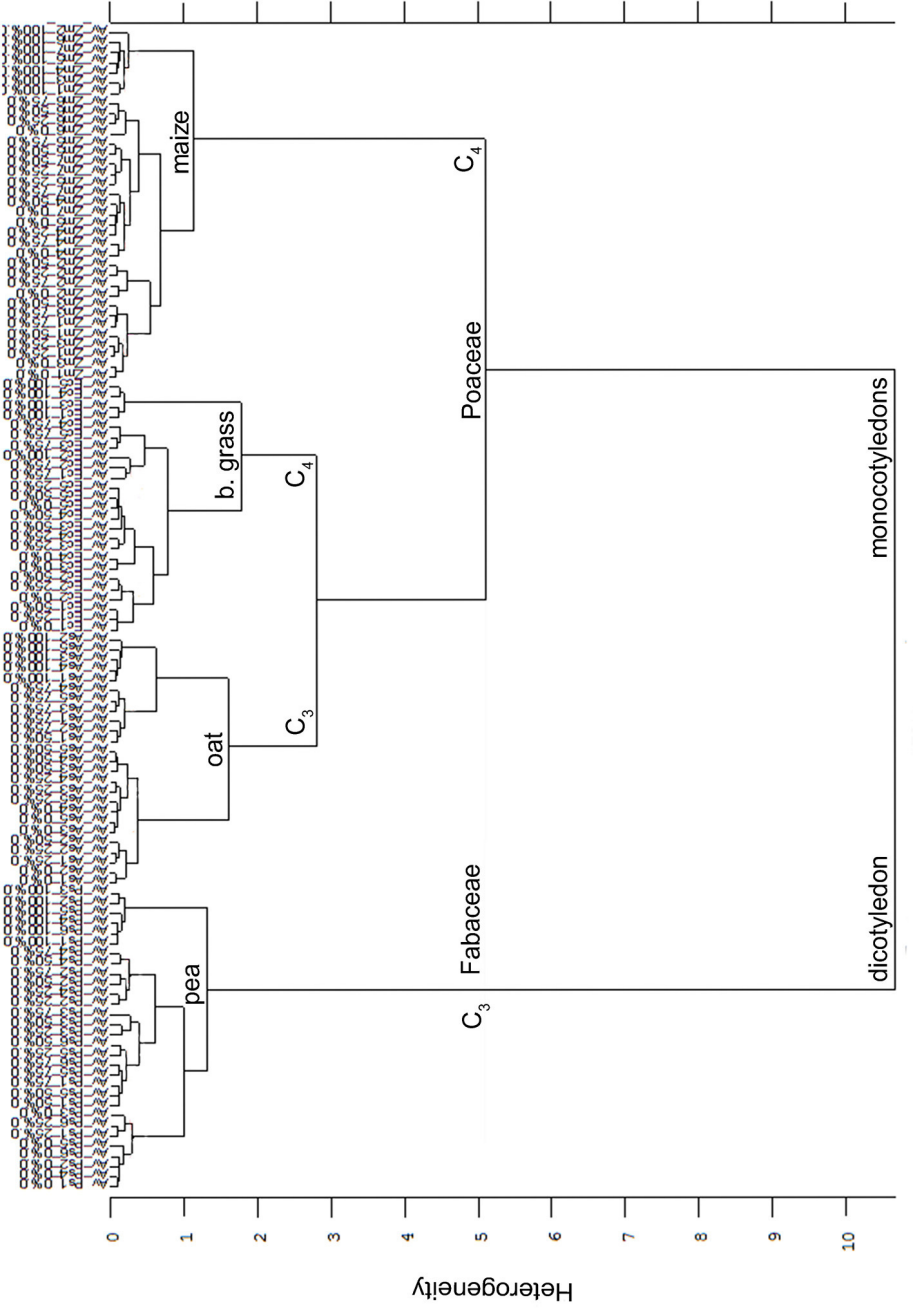
Cluster analysis of the Av-spectra of all cultivars of the species *Pisum sativum* (Ps, pea), *Avena sativa* (As, oat), *Zea mays* (Zm, maize) and all proveniences of the species *Echinochloa crus-galli* (Ec, barnyard grass) for the united experiment 1 (exp. 1-1 and 1-2) as well as the united experiment 2 (exp. 2-1 and 2-2). Cluster analysis was evaluated with the first derivation and vector normalization of the reduced frequency range (3,751–2,749 cm^−1^ and 1,801–599 cm^−1^), Ward's algorithm and Euclidian distance. The Av-spectra are averages (Av) of FTIR-ATR spectra of the different root segments (100% root tip, 0% root basis etc.; see Figure 1 for scheme, Table 2 for n, Table 1 for names of cultivars and proveniences).

Secondly, summarizing all Av-spectra of the four experiments with all species, cultivars and proveniences in one cluster analysis (Figure 7), the root segments of the dicotyledon pea were clearly separated from the root segments of the monocotyledons oat, barnyard grass and maize. This interspecific heterogeneity was with 10.67 very high. Within the Poaceae, maize roots were separated at heterogeneity of 5.11 from the roots of oat and barnyard grass, whereas the last two were separated from each other at the heterogeneity of 2.81. The partitioning of the roots of the mono- and dicotyledons carried more weight than the C_3_/C_4_ discrimination. The intraspecific heterogeneity was low in maize (1.14), pea (1.32), oat (1.60) and barnyard grass (1.78). Considering Figure 7 in detail, it was not possible to separate the cultivars and proveniences within the species but remarkably, the 0 and 100% root spectra clustered more or less precisely around one another.

In summary, the selection of the cultivar or provenience of the species pea, oat, maize and barnyard grass had no influence on the differentiability of the species to each other.

### Cultivar differentiation

**Figure 8:**
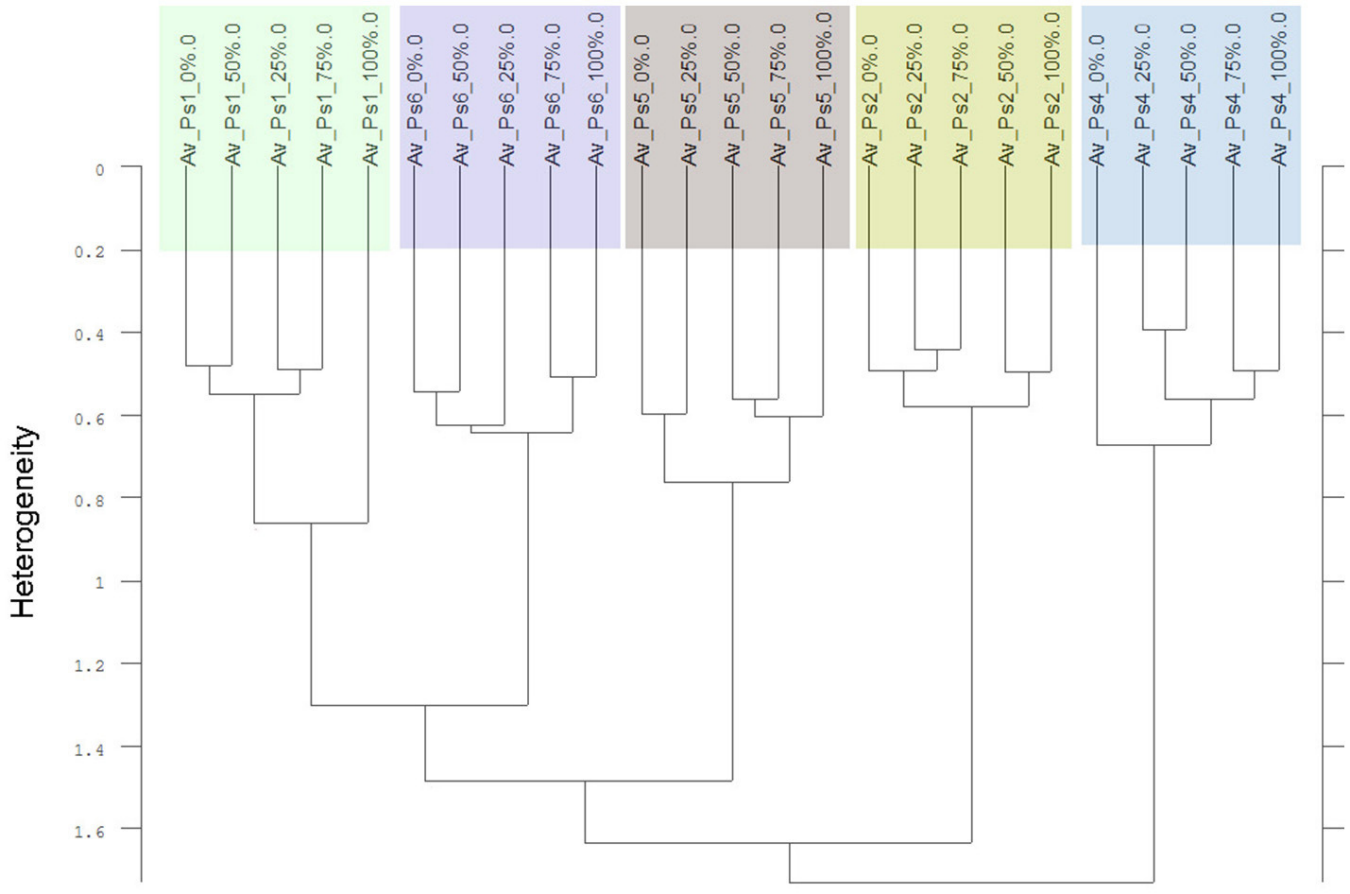
Cluster analysis of the Av-spectra of five of the six cultivars of the species *Pisum sativum* (Ps, pea) of the experiment 1-1, omission of cultivar 3 (Ps3). Complete frequency range (3,997–374 cm^−1^) of the Av-spectra was evaluated with the second derivation and vector normalization, Ward's algorithm und Euclidian distance. The Av-spectra are averages (Av) of FTIR-ATR spectra of the different root segments (100% root tip, 0% root basis etc.; see Figure 1 for scheme, Table 2 for n, Table 1 for names of cultivars and proveniences).

**Figure 9:**
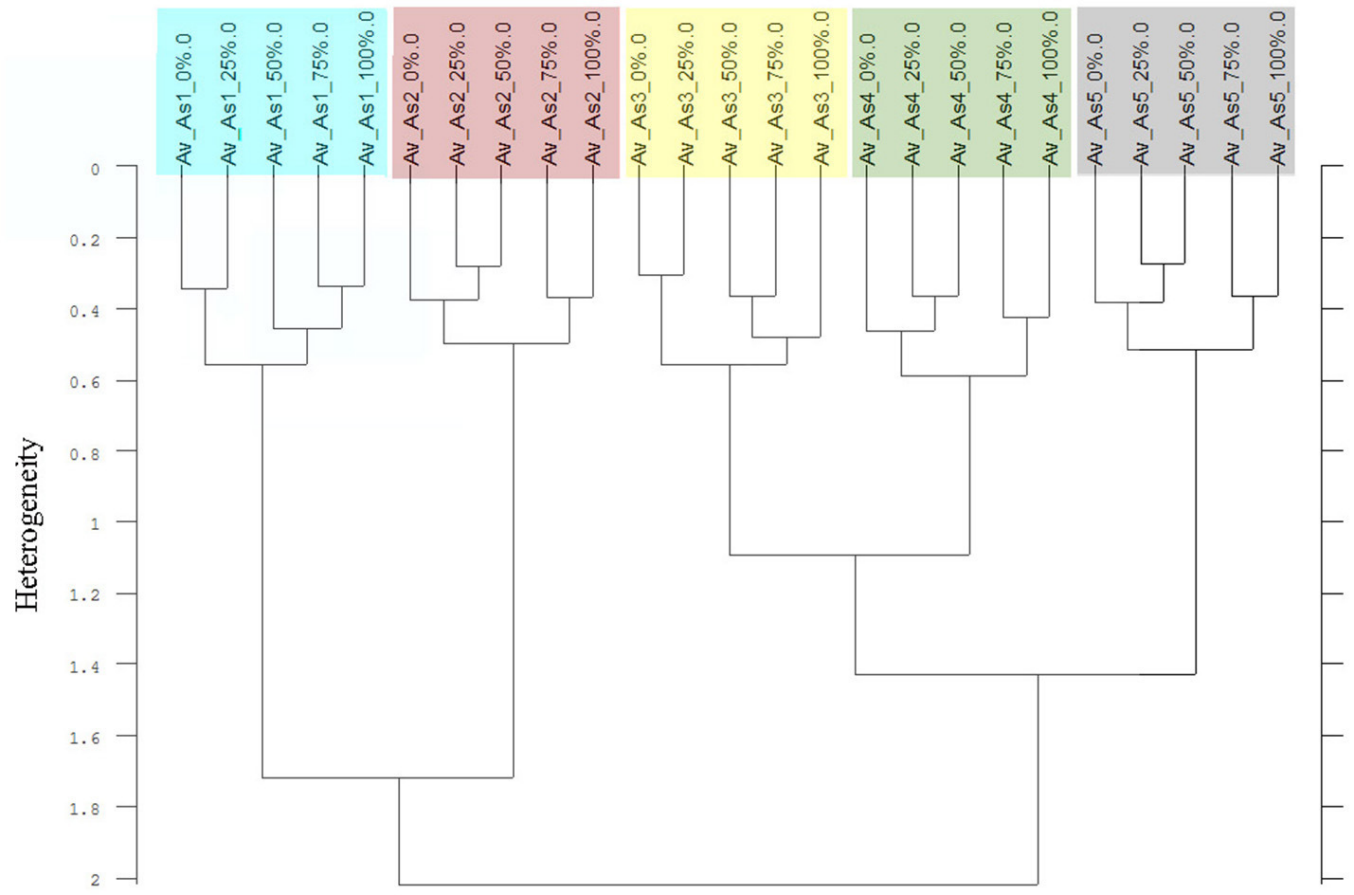
Cluster analysis of the Av-spectra of all cultivars of the species *Avena sativa* (As, oat) for the experiment 1-1. The complete frequency range (3,997–374 cm^−1^) of the Av-spectra was evaluated with the second derivation and vector normalization, Ward's algorithm und Euclidian distance. The Av-spectra are averages (Av) of FTIR-ATR spectra of the different root segments (100% root tip, 0% root basis etc.; see Figure 1 for scheme, Table 2 for n, Table 1 for names of cultivars and proveniences).

**Figure 10:**
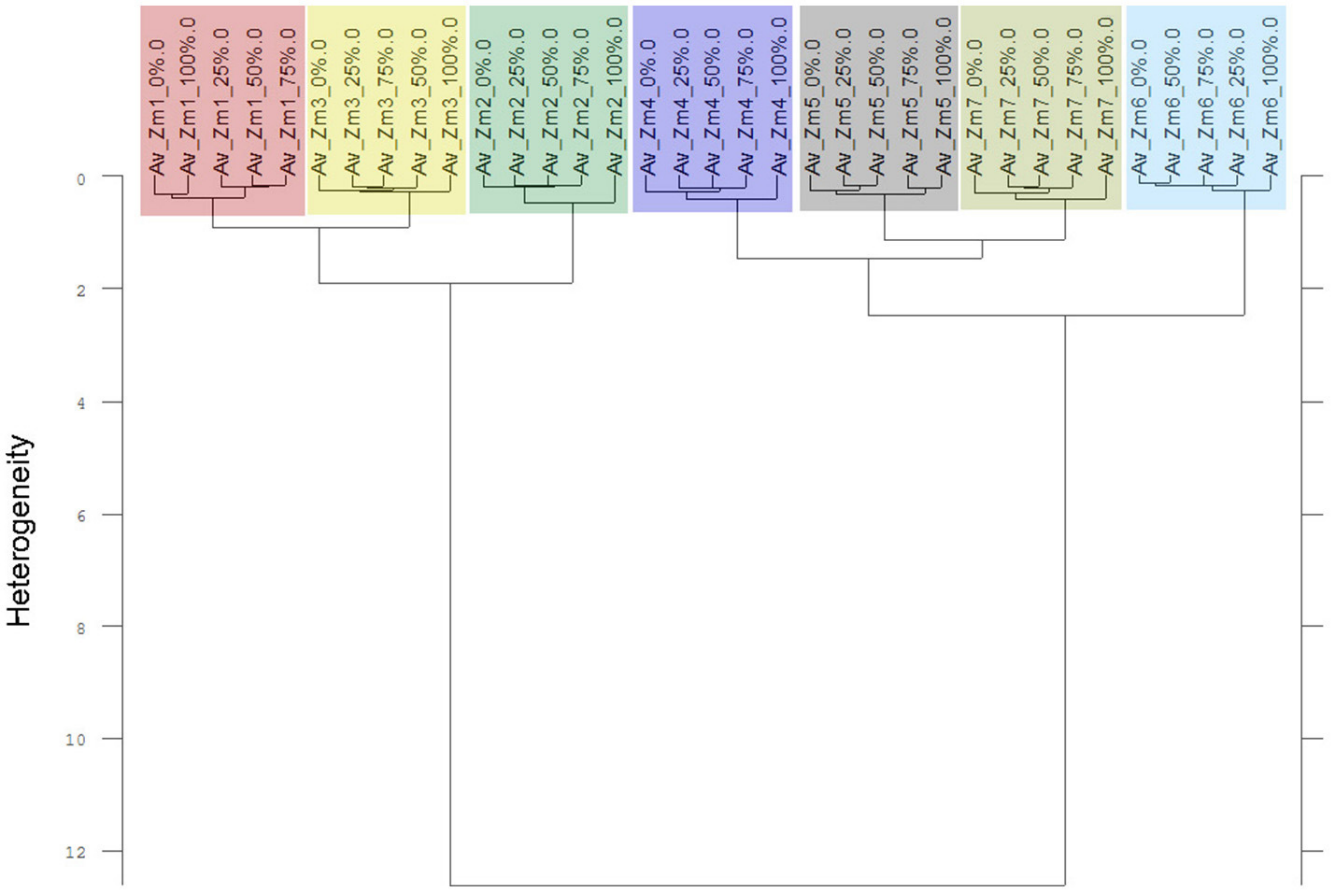
Cluster analysis of the Av-spectra of all cultivars of the species *Zea mays* (Zm, maize) for the experiment 2-2. The reduced frequency range (3,751–2,749 cm^−1^ and 1,801–599 cm^−1^) of the Av-spectra was evaluated with the second derivation and vector normalization, Ward's algorithm und Euclidian distance. The Av-spectra are averages (Av) of FTIR-ATR spectra of the different root segments (100% root tip, 0% root basis etc.; see Figure 1 for scheme, Table 2 for n, Table 1 for names of cultivars and proveniences).

**Figure 11:**
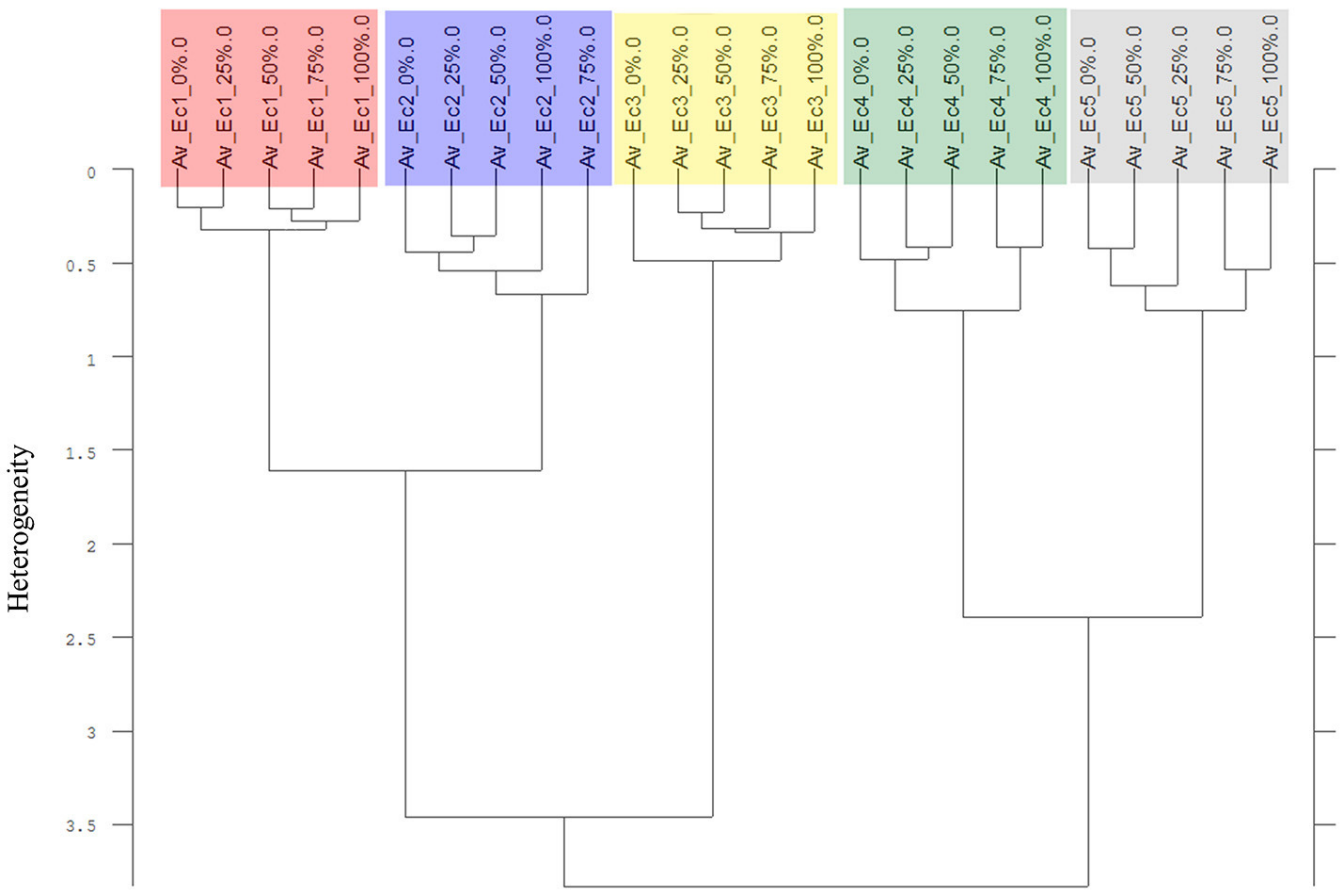
Cluster analysis of the Av-spectra of all proveniences of the species *Echinochloa crus-galli* (Ec, barnyard grass) for the experiment 2-2. Complete frequency range (3,997–374 cm^−1^) of the Av-spectra was evaluated with the second derivation and vector normalization, Ward's algorithm und Euclidian distance. The Av-spectra are averages (Av) of FTIR-ATR spectra of the different root segments (100% root tip, 0% root basis etc.; see Figure 1 for scheme, Table 2 for n, Table 1 for names of cultivars and proveniences).

Thirdly, in two of the four experiments a differentiation of the individual cultivars and proveniences was possible. Considering only the Av-spectra of the pea cultivars of experiment 1-1 in a cluster analysis, three of the cultivars split up (Supplementary Figure 2). For that separation only the usage of the complete spectral range led to a result. The wrinkled pea (Ps4), the semi-leafless cultivar (Ps2) and the field pea (Ps5) were separated in own clusters, the remaining three were mixed. A cluster analysis of Av-spectra of pea without the third cultivar (Ps3, winter pea) led to a separation of all remaining cultivars (Figure 8). The leafed pea (Ps1) and sugar pea (Ps6) formed a cluster with the heterogeneity of 1.30. These two formed with the field pea (Ps5) a cluster with the heterogeneity of 1.48. Together with the semi-leafless pea (Ps2) they formed a cluster with the heterogeneity of 1.64. The wrinkled pea (Ps4) splitted from the other pea cultivars at the heterogeneity of 1.72. The heterogeneity in general was low and remarkably, the pea cultivars did not form bigger groups as it is seen in Figures 9–11. The oat Av-spectra of experiment 1-1 were also divided into cultivar-clusters (Figure 9). Here, the white (As1) and yellow oat (As2) formed a cluster with heterogeneity of 1.72 and the remaining three cultivars split up at heterogeneity of 1.43 where the winter oat (As5) was separated from black oat (As3) and hulless oat (As4). Again, the heterogeneity in general was low.

The Av-spectra of maize of experiment 2-2 were analyzed with a cluster analysis and revealed a separation of all cultivars (Figure 10). Here, a reduced spectral range (3,751–2,749 cm^−1^ and 1,801–599 cm^−1^) was used because the application of the complete spectral range classified the 0% Av-spectra (root basis) of Zm5 to the Zm4-cluster. The cultivars split at the high heterogeneity of 12.62 in two big clusters. Flint maize (Zm1), dent maize (Zm3) and popcorn maize (Zm2) separated with the low heterogeneity of 1.90 from each other whereas sweet corn (Zm4), black pod corn (Zm6), early (Zm5) and late ripening (Zm7) cultivars separated from each other at the heterogeneity of 2.47. The Av-spectra of barnyard grass of the same experiment (2-2) also clustered into all of their proveniences (Figure 11) when performing a cluster analysis with the complete spectral range. The two big groups separated at a low heterogeneity (3.83). The first group consisted of barnyard grass from Germany (Ec1), Greece (Ec2) and Canada (Ec3). Ec3 was divided from Ec1 and Ec2 at 3.46, whereas Ec1 and Ec2 split at 1.61. Barnyard grass from China (Ec4) and Poland (Ec5) where the second big group which divided at heterogeneity of 2.39 (Figure 11).

Contrary to the species differentiation, the differentiation of the cultivars and proveniences was only possible with the data processing “second derivation” and had only low heterogeneities (except maize which had a high heterogeneity). Additionally, these results could not be replicated in all experiments. Therefore, the separation of the cultivars and proveniences had less conclusive results and was less resilient.

## Discussion

### Species differentiation

The present work demonstrated that roots of pea, oat, maize and barnyard grass could be differentiated with FTIR spectroscopy and cluster analyses independent of the tested cultivar or proveniences of the species. It was already proven that the roots of pea and oat as well as of maize and barnyard grass could be clearly separated with FTIR spectroscopy (Naumann et al., 2010; Meinen and Rauber, 2015). These studies used only one cultivar per species. The heterogeneity of all cluster analyses in the present work was high which gives evidence of the high accuracy of this method and analysis. Furthermore, the experiments were replicated at different points in time and showed only slight differences in the results of the evaluation. Additionally, we used next to the seminal root also the first lateral root or the adventitious root. There were only minor spectral differences between the different types of roots which did not influence the results of the cluster analysis and thus, all were included in the analyses. Considering the root segments, each root segment was equally qualified for the species differentiation. In experiment 1-2, two oat cultivars (As2, As4) could not be discriminated from the pea cultivars. This non-separation was caused by the root tip spectra (100% Av-spectra) and could be prevented by the exclusion of the root tip spectra. The same was true for some non-separations found in the experiments 2-1 and 2-2 with maize and barnyard grass. With the exclusion of the root tip spectra, each cultivar of maize could be separated clearly from each provenience of barnyard grass. This is in accordance with Meinen and Rauber (2015) who had to remove root tips of wheat and blackgrass to get a species differentiation by FTIR spectroscopy. As the root tip is a zone of continuous cell proliferation and differentiation, these cells could be too similar in their chemical composition to be distinguishable in the cluster analysis. When the cells are more differentiated in subsequent parts of the root, the cluster analysis could separate the species without failure. Nevertheless, Zhao et al. (2004) tested ground root tips of eight wheat cultivars with FTIR spectroscopy and could differentiate between those cultivars. This is contrary to our results which proved the root tips to be undifferentiated in some cases.

The pea spectra could be clearly separated from the oat spectra, when comparing all cultivars of pea with all cultivars of oat. This could also be clearly shown for maize and barnyard grass. As reported by Naumann et al. (2010) and Meinen and Rauber (2015), the interspecific heterogeneity is much higher than the intraspecific heterogeneity in pea and oat as well as in maize and barnyard grass. The chemical composition of roots seems to be species dependent and therefore typical for a certain species. Zeier and Schreiber (1998) investigated endodermal cell walls of five monocotyledonous species by chemical and chromatographic analyses and found species specific differences. The chemical composition of cells can be recorded by FTIR spectroscopy and the according spectra can be used as spectral fingerprint in root analyses. All used species showed a clear peak in the wavenumber range between 3,700 and 3,000 cm^−1^ which is dominated by the presence of various functional groups (O-H and N-H) and between 3,000 and 2,800 cm^−1^ which displays the C-H stretching region with lipids, wax and fats. The wavenumbers 2,920 and 2,850 cm^−1^ are specific for lipids (Artz et al., 2008). In that wavenumber range, the Av-spectra show differences between the species. The more important peaks for species discrimination appeared between 1,800 and 1,500 cm^−1^, this wavenumber range is dominated by proteins, especially the wavenumber ranges between 1,653 and 1,600 cm^−1^, followed by a protein/lipid section with the wavenumber range between 1,500 and 1,200 cm^−1^. Again, all used FTIR spectra show species specific differences between the peak intensities in this region. The highest peak of the spectra was shown ~1,000 cm^−1^. This peak is assigned to the cellulose and hemicellulose and showed also species specific values. All these peaks are in accordance to various publications and are known to be species specific (Artz et al., 2008; Naumann, 2009; Naumann et al., 2010; Rewald et al., 2012; Meinen and Rauber, 2015). The FTIR spectroscopy is thus a promising tool for further root analyses in plant mixtures. FTIR spectroscopy irradiates the sample with mid-infrared light waves only in a few micrometers in depth (Chalmers and Griffiths, 2017). Therefore, the chemical composition of the outer root layers is measured. Nevertheless, species differentiation is possible using dried root segments, even in closely related species like maize and barnyard grass. This finding confirms the results of Meinen and Rauber (2015).

The clear discrimination between all four species showed that the differentiation of mono- and dicotyledons carried more weight than the C_3_/C_4_ differentiation. This was already shown with FTIR root spectra of crop and weed species, e.g., maize/barnyard grass (Meinen and Rauber, 2015) and with FTIR leaf spectra of different plant species (Kim et al., 2004). Equally, the substantial separation occurred between mono- and dicotyledons and the subgroups consisted of C_3_ and C_4_ species. Schreiber et al. (1999) analyzed the chemical composition of endodermal and hypodermal cell walls of seven mono- and three dicotyledonous plant species. They found that lignin contents in the Casparian stripes of the monocotyledonous species *Monstera deliciosa* and *Clivia miniata* were higher than in the dicotyledonous species *Pisum sativum*. Additionally to this result, more chemical differences in the endodermal and hypodermal cell walls between species and especially between mono- and dicotyledonous species were detected by the analyzation of the chemical composition of the cell walls.

### Cultivar differentiation

Besides the species differentiation, it was also possible to show in two of the four experiments a differentiation of cultivars within the four species. Pea, oat (both exp. 1-1) and barnyard grass (exp. 2-2) showed only a cultivar differentiation while using the complete spectral range and the second derivation evaluation. For maize (exp. 2-2), only the reduced spectral range and the second derivation evaluation led to the cultivar differentiation. The cultivar differentiation shown here has to be regarded with caution because of the following reasons: The heterogeneity of the cluster analyses was very low (except maize). The results could not be replicated in the second experiment and the evaluation process had to be adapted in comparison to all other evaluations undertaken for this study. In the other two experiments which could not be used for the cultivar differentiation, the recorded spectra were somewhat more heterogeneous. This difference did not carry so much weight that the species differentiation was affected but yet so much that the cultivar differentiation was no longer possible. It was not evident why the Av-spectra were different in the experiments. Both experiments (1-1 and 2-2) did not take place at the same time and neither substrate or weather/water conditions were different. There is no obvious difference in the spectra but a slightly higher heterogeneity.

For the species pea, the pea cultivar 3 (Ps3, EFB 33) had to be omitted that the remaining five pea cultivar could be differentiated accurately. EFB 33 is a cultivar which is not listed in the German “Descriptive Variety Lists.” The feature of this cultivar could possibly be unsteady in the generation times which may cause heterogeneous Av-spectra leading to the non-differentiation of this cultivar. The cluster analysis of the maize cultivars has a higher heterogeneity (12.62) than the other cluster analyses and the heterogeneity within each cultivar is very low (between 0.26 and 0.47) pointing to a more reliable result. The reason of this could possibly be that maize breeding process from teosinte (*Zea mays ssp. mexicana* L.) to the crop maize (*Zea mays ssp. mays* L.) is much stronger than the breeding process for pea or oat and has therefore strong genetic features which also influence the spectra of the roots.

## Conclusion

The dried root segments of each cultivar of pea were distinguishable from the dried roots segments of each cultivar of oat (1:1 comparison) as well as each cultivar of maize from each provenience of barnyard grass. In some cases the Av-spectra of the root tips were more heterogeneous which disturbed the differentiability and these spectra had to be omitted to ensure successful 1:1 comparison. Thus, the root spectra were species specific, but independent of the cultivars. This was found in the far related species pea and oat and even in the closely related species maize and barnyard grass. The differentiation was also possible with all cultivars of pea and oat within one cluster analysis. This was also true for maize and barnyard grass. Consequently, in further studies, cultivar choice should not interfere with species differentiation using FTIR spectroscopy. Bringing all cultivars and proveniences of the four species in one cluster analysis, the species could be separated from each other whereas pea as a dicotyledonous plant separated clearly from the monocotyledonous oat, barnyard grass and maize. Additionally, we found in two of the four experiments a differentiation of the cultivar within a species. This differentiation is difficult to replicate and depends on various unknown criteria and therefore has to be verified in further experiments. As the already published experiments only used one cultivar/provenience of the species, we ensured with our experiments the reliability for more than one cultivar/provenience. This can be the basis for further experiments which investigate the response of roots to different factors, e.g., drought, pest attack, plant diseases or elevated CO_2_ concentration.

## Author contributions

NL, CM, and RR developed the study design. NL conducted the research. NL with the support of CM and RR conducted the analysis and NL, CM, and RR wrote the paper.

### Conflict of interest statement

The authors declare that the research was conducted in the absence of any commercial or financial relationships that could be construed as a potential conflict of interest.
