# Supplementary material for: Root Differentiation of Agricultural Plant Cultivars and Proveniences Using FTIR Spectroscopy
Source: Front Plant Sci. 2018 Jun 5;9:748. doi: 10.3389/fpls.2018.00748 (PMC6008560; doi:10.3389/fpls.2018.00748)
Supplement: Supplementary file 1 [file Data_Sheet_1.DOCX]

**Supplementary Figue 1A-D:** FTIR spectra measured of ten root samples of the middle section of the root (50%). Displayed are pea (A, cultivar Ps1), oat (B, cultivar As1), maize (C, cultivar Zm1), and barnyard grass (D, cultivar Ec1) in blue and the corresponding Av-spectra in red. Spectra are vector-normalized and offset-corrected.

**Supplementary Figure 2**: Cluster analysis of the Av-spectra of the different cultivars of the species *Pisum sativum* (Ps, pea) of experiment 1-1. The complete frequency range (3997-374 cm^-1^) of the Av-spectra was evaluated with the second derivation and vector normalization, Ward’s algorithm und Euclidian distance. The Av-spectra are averages (Av) of FTIR-ATR spectra of the different root segments (100 % root tip, 0 % root basis etc.; see Fig. 1 for scheme, Tab. 2 for n, Tab. 1 for names of cultivars and proveniences).
