# Supplementary material for: Root Differentiation of Agricultural Plant Cultivars and Proveniences Using FTIR Spectroscopy
Source: Front Plant Sci. 2018 Jun 5;9:748. doi: 10.3389/fpls.2018.00748 (PMC6008560; doi:10.3389/fpls.2018.00748)
Supplement: Supplementary file 2 [file Data_Sheet_2.docx]

**Supplementary Table 1:** Heterogeneity of the results of the cluster analyses of the 1:1 comparisons for experiment 1-1, 1-2 and united 1. The reduced frequency range (3751-2749 cm^-1^ and 1801-599 cm^‑1^) of the Av-spectra was evaluated with the second derivation and vector normalization, Ward’s algorithm und Euclidian distance. Abbreviations: Ps *Pisum sativum* (pea), As *Avena sativa* (oat). The heterogeneity “all” stands for the heterogeneity where the species split. * stands for a cluster analysis without the Av_100 % spectra.

| **pea - oat (exp. 1-1)** | All | Pea | Oat |
| --- | --- | --- | --- |
| (n=5) Ps1 - As1 (n=5) | 1.34 | 0.49 | 0.43 |
| (n=5) Ps1 - As2 (n=5) | 1.48 | 0.49 | 0.34 |
| (n=5) Ps1 - As3 (n=5) | 1.40 | 0.49 | 0.49 |
| (n=5) Ps1 - As4 (n=5) | 1.32 | 0.49 | 0.46 |
| (n=5) Ps1 - As5 (n=5) | 1.37 | 0.49 | 0.36 |
|  |  |  |  |
| (n=5) Ps2 - As1 (n=5) | 1.38 | 0.45 | 0.43 |
| (n=5) Ps2 - As2 (n=5) | 1.54 | 0.45 | 0.34 |
| (n=5) Ps2 - As3 (n=5) | 1.38 | 0.45 | 0.49 |
| (n=5) Ps2 - As4 (n=5) | 1.34 | 0.45 | 0.46 |
| (n=5) Ps2 - As5 (n=5) | 1.35 | 0.45 | 0.36 |
|  |  |  |  |
| (n=5) Ps3 - As1 (n=5) | 1.47 | 0.47 | 0.43 |
| (n=5) Ps3 - As2 (n=5) | 1.62 | 0.47 | 0.34 |
| (n=5) Ps3 - As3 (n=5) | 1.54 | 0.47 | 0.49 |
| (n=5) Ps3 - As4 (n=5) | 1.43 | 0.47 | 0.46 |
| (n=5) Ps3 - As5 (n=5) | 1.49 | 0.47 | 0.36 |
|  |  |  |  |
| (n=5) Ps4 - As1 (n=5) | 1.35 | 0.39 | 0.43 |
| (n=5) Ps4 - As2 (n=5) | 1.51 | 0.39 | 0.34 |
| (n=5) Ps4 - As3 (n=5) | 1.39 | 0.39 | 0.49 |
| (n=5) Ps4 - As4 (n=5) | 1.34 | 0.39 | 0.46 |
| (n=5) Ps4 - As5 (n=5) | 1.35 | 0.39 | 0.36 |
|  |  |  |  |
| (n=5) Ps5 - As1 (n=5) | 1.35 | 0.50 | 0.43 |
| (n=5) Ps5 - As2 (n=5) | 1.49 | 0.50 | 0.34 |
| (n=5) Ps5 - As3 (n=5) | 1.43 | 0.50 | 0.49 |
| (n=5) Ps5 - As4 (n=5) | 1.35 | 0.50 | 0.46 |
| (n=5) Ps5 - As5 (n=5) | 1.41 | 0.50 | 0.36 |
|  |  |  |  |
| (n=5) Ps6 - As1 (n=5) | 1.32 | 0.40 | 0.43 |
| (n=5) Ps6 - As2 (n=5) | 1.43 | 0.40 | 0.34 |
| (n=5) Ps6 - As3 (n=5) | 1.35 | 0.40 | 0.49 |
| (n=5) Ps6 - As4 (n=5) | 1.28 | 0.40 | 0.46 |
| (n=5) Ps6 - As5 (n=5) | 1.30 | 0.40 | 0.36 |
|  |  |  |  |
|  |  |  |  |
| **pea - oat (exp. 1-2)** | All | Pea | Oat |
| (n=5) Ps1 - As1 (n=5) | 1.28 | 0.39 | 0.40 |
| (n=5) Ps1 - As2 (n=4) * | 1.50 | 0.39 | 0.28 |
| (n=5) Ps1 - As3 (n=5) | 1.50 | 0.39 | 0.40 |
| (n=5) Ps1 - As4 (n=4) * | 1.52 | 0.39 | 0.32 |
| (n=5) Ps1 - As5 (n=5) | 1.41 | 0.39 | 0.54 |
|  |  |  |  |
| (n=5) Ps2 - As1 (n=5) | 1.20 | 0.59 | 0.40 |
| (n=5) Ps2 - As2 (n=4) * | 1.37 | 0.59 | 0.28 |
| (n=5) Ps2 - As3 (n=5) | 1.38 | 0.59 | 0.40 |
| (n=5) Ps2 - As4 (n=4) * | 1.36 | 0.59 | 0.32 |
| (n=5) Ps2 - As5 (n=5) | 1.30 | 0.59 | 0.54 |
|  |  |  |  |
| (n=5) Ps3 - As1 (n=5) | 1.62 | 0.42 | 0.40 |
| (n=5) Ps3 - As2 (n=5) | 1.71 | 0.42 | 0.61 |
| (n=5) Ps3 - As3 (n=5) | 1.83 | 0.42 | 0.40 |
| (n=5) Ps3 - As4 (n=5) | 1.73 | 0.42 | 0.53 |
| (n=5) Ps3 - As5 (n=5) | 1.70 | 0.42 | 0.54 |
|  |  |  |  |
| (n=5) Ps4 - As1 (n=5) | 1.34 | 0.43 | 0.40 |
| (n=5) Ps4 - As2 (n=5) | 1.40 | 0.43 | 0.61 |
| (n=5) Ps4 - As3 (n=5) | 1.41 | 0.43 | 0.40 |
| (n=5) Ps4 - As4 (n=4) * | 1.37 | 0.43 | 0.32 |
| (n=5) Ps4 - As5 (n=5) | 1.33 | 0.43 | 0.54 |
|  |  |  |  |
| (n=5) Ps5 - As1 (n=5) | 1.44 | 0.46 | 0.40 |
| (n=5) Ps5 - As2 (n=4) * | 1.65 | 0.46 | 0.28 |
| (n=5) Ps5 - As3 (n=5) | 1.68 | 0.46 | 0.40 |
| (n=5) Ps5 - As4 (n=4) * | 1.69 | 0.46 | 0.32 |
| (n=5) Ps5 - As5 (n=5) | 1.55 | 0.46 | 0.54 |
|  |  |  |  |
| (n=5) Ps6 - As1 (n=5) | 1.44 | 0.39 | 0.40 |
| (n=5) Ps6 - As2 (n=4) * | 1.67 | 0.39 | 0.28 |
| (n=5) Ps6 - As3 (n=5) | 1.68 | 0.39 | 0.40 |
| (n=5) Ps6 - As4 (n=4) * | 1.71 | 0.39 | 0.32 |
| (n=5) Ps6 - As5 (n=5) | 1.56 | 0.39 | 0.54 |
|  |  |  |  |
|  |  |  |  |
| **pea - oat (united exp. 1)** | All | Pea | Oat |
| (n=5) Ps1 - As1 (n=5) | 1.34 | 0.34 | 0.40 |
| (n=5) Ps1 - As2 (n=5) | 1.48 | 0.34 | 0.35 |
| (n=5) Ps1 - As3 (n=5) | 1.42 | 0.34 | 0.45 |
| (n=5) Ps1 - As4 (n=5) | 1.38 | 0.34 | 0.44 |
| (n=5) Ps1 - As5 (n=5) | 1.42 | 0.34 | 0.35 |
|  |  |  |  |
| (n=5) Ps2 - As1 (n=5) | 1.34 | 0.49 | 0.40 |
| (n=5) Ps2 - As2 (n=5) | 1.49 | 0.49 | 0.35 |
| (n=5) Ps2 - As3 (n=5) | 1.38 | 0.49 | 0.45 |
| (n=5) Ps2 - As4 (n=5) | 1.33 | 0.49 | 0.44 |
| (n=5) Ps2 - As5 (n=5) | 1.35 | 0.49 | 0.35 |
|  |  |  |  |
| (n=5) Ps3 - As1 (n=5) | 1.52 | 0.34 | 0.40 |
| (n=5) Ps3 - As2 (n=5) | 1.66 | 0.34 | 0.35 |
| (n=5) Ps3 - As3 (n=5) | 1.64 | 0.34 | 0.45 |
| (n=5) Ps3 - As4 (n=5) | 1.56 | 0.34 | 0.44 |
| (n=5) Ps3 - As5 (n=5) | 1.60 | 0.34 | 0.35 |
|  |  |  |  |
| (n=5) Ps4 - As1 (n=5) | 1.40 | 0.34 | 0.40 |
| (n=5) Ps4 - As2 (n=5) | 1.56 | 0.34 | 0.35 |
| (n=5) Ps4 - As3 (n=5) | 1.44 | 0.34 | 0.45 |
| (n=5) Ps4 - As4 (n=5) | 1.38 | 0.34 | 0.44 |
| (n=5) Ps4 - As5 (n=5) | 1.40 | 0.34 | 0.35 |
|  |  |  |  |
| (n=5) Ps5 - As1 (n=5) | 1.40 | 0.42 | 0.40 |
| (n=5) Ps5 - As2 (n=5) | 1.53 | 0.42 | 0.35 |
| (n=5) Ps5 - As3 (n=5) | 1.53 | 0.42 | 0.45 |
| (n=5) Ps5 - As4 (n=5) | 1.46 | 0.42 | 0.44 |
| (n=5) Ps5 - As5 (n=5) | 1.49 | 0.42 | 0.35 |
|  |  |  |  |
| (n=5) Ps6 - As1 (n=5) | 1.34 | 0.35 | 0.40 |
| (n=5) Ps6 - As2 (n=5) | 1.50 | 0.35 | 0.35 |
| (n=5) Ps6 - As3 (n=5) | 1.49 | 0.35 | 0.45 |
| (n=5) Ps6 - As4 (n=5) | 1.41 | 0.35 | 0.44 |
| (n=5) Ps6 - As5 (n=5) | 1.44 | 0.35 | 0.35 |

**Supplementary Table 2:** Heterogeneity of the results of the cluster analyses of the 1:1 comparisons for experiment 2-1, 2-2 and united 2. The reduced frequency range (3751-2749 cm^-1^ and 1801-599 cm^‑1^) of the Av-spectra was evaluated with the second derivation and vector normalization, Ward’s algorithm und Euclidian distance. Abbreviations: Zm *Zea mays* (maize), Ec *Echinochloa crus‑galli* (barnyard grass). The heterogeneity “all” stands for the heterogeneity where the species split. – stands for the non-germination of Ec2. * stands for a cluster analysis without the Ec_100% spectra. ** stands for a cluster analysis without both 100% spectra (Zm_100% and Ec_100%).

| **maize - b. grass (exp. 2-1)** | All | Maize | B. grass |
| --- | --- | --- | --- |
| (n=5) Zm1 - Ec1 (n=5) | 0.99 | 0.33 | 0.79 |
| (n=5) Zm1 - Ec2 (n=5) | - | - | - |
| (n=5) Zm1 - Ec3 (n=5) | 0.89 | 0.33 | 0.67 |
| (n=5) Zm1 - Ec4 (n=5) | 0.90 | 0.33 | 0.73 |
| (n=5) Zm1 - Ec5 (n=5) | 1.01 | 0.33 | 0.66 |
|  |  |  |  |
| (n=5) Zm2 - Ec1 (n=4) * | 0.73 | 0.47 | 0.33 |
| (n=5) Zm2 - Ec2 (n=5) | - | - | - |
| (n=5) Zm2 - Ec3 (n=4) * | 0.66 | 0.47 | 0.29 |
| (n=4) Zm2 - Ec4 (n=4) ** | 0.64 | 0.37 | 0.43 |
| (n=5) Zm2 - Ec5 (n=4) * | 0.77 | 0.47 | 0.30 |
|  |  |  |  |
| (n=5) Zm3 - Ec1 (n=5) | 1.15 | 0.40 | 0.79 |
| (n=5) Zm3 - Ec2 (n=5) | - | - | - |
| (n=5) Zm3 - Ec3 (n=5) | 1.02 | 0.40 | 0.67 |
| (n=5) Zm3 - Ec4 (n=5) | 1.01 | 0.40 | 0.73 |
| (n=5) Zm3 - Ec5 (n=5) | 1.14 | 0.40 | 0.66 |
|  |  |  |  |
| (n=5) Zm4 - Ec1 (n=5) | 0.94 | 0.28 | 0.79 |
| (n=5) Zm4 - Ec2 (n=5) | - | - | - |
| (n=5) Zm4 - Ec3 (n=5) | 0.82 | 0.28 | 0.67 |
| (n=5) Zm4 - Ec4 (n=5) | 0.81 | 0.28 | 0.73 |
| (n=5) Zm4 - Ec5 (n=5) | 0.93 | 0.28 | 0.66 |
|  |  |  |  |
| (n=5) Zm5 - Ec1 (n=4) * | 0.75 | 0.31 | 0.33 |
| (n=5) Zm5 - Ec2 (n=5) | - | - | - |
| (n=5) Zm5 - Ec3 (n=4) * | 0.64 | 0.31 | 0.29 |
| (n=5) Zm5 - Ec4 (n=4) * | 0.67 | 0.31 | 0.43 |
| (n=5) Zm5 - Ec5 (n=5) | 0.83 | 0.31 | 0.66 |
|  |  |  |  |
| (n=5) Zm6 - Ec1 (n=5) | 0.88 | 0.31 | 0.79 |
| (n=5) Zm6 - Ec2 (n=5) | - | - | - |
| (n=5) Zm6 - Ec3 (n=5) | 0.76 | 0.31 | 0.67 |
| (n=5) Zm6 - Ec4 (n=4) * | 0.82 | 0.31 | 0.43 |
| (n=5) Zm6 - Ec5 (n=5) | 0.88 | 0.31 | 0.66 |
|  |  |  |  |
| (n=5) Zm7 - Ec1 (n=5) | 0.94 | 0.38 | 0.79 |
| (n=5) Zm7 - Ec2 (n=5) | - | - | - |
| (n=5) Zm7 - Ec3 (n=5) | 0.83 | 0.38 | 0.67 |
| (n=5) Zm7 - Ec4 (n=4) * | 0.72 | 0.38 | 0.43 |
| (n=5) Zm7 - Ec5 (n=5) | 0.95 | 0.38 | 0.66 |
|  |  |  |  |
|  |  |  |  |
| **maize - b. grass (exp. 2-2)** | All | Maize | B. grass |
| (n=5) Zm1 - Ec1 (n=5) | 2.61 | 0.20 | 0.32 |
| (n=5) Zm1 - Ec2 (n=5) | 1.81 | 0.20 | 0.42 |
| (n=5) Zm1 - Ec3 (n=5) | 1.65 | 0.20 | 0.46 |
| (n=5) Zm1 - Ec4 (n=5) | 1.54 | 0.20 | 0.52 |
| (n=5) Zm1 - Ec5 (n=5) | 1.42 | 0.20 | 0.53 |
|  |  |  |  |
| (n=5) Zm2 - Ec1 (n=5) | 2.70 | 0.48 | 0.32 |
| (n=5) Zm2 - Ec2 (n=5) | 1.68 | 0.48 | 0.42 |
| (n=5) Zm2 - Ec3 (n=5) | 1.60 | 0.48 | 0.46 |
| (n=5) Zm2 - Ec4 (n=5) | 1.39 | 0.48 | 0.52 |
| (n=5) Zm2 - Ec5 (n=4) * | 1.19 | 0.48 | 0.24 |
|  |  |  |  |
| (n=5) Zm3 - Ec1 (n=5) | 2.74 | 0.21 | 0.32 |
| (n=5) Zm3 - Ec2 (n=5) | 1.92 | 0.21 | 0.42 |
| (n=5) Zm3 - Ec3 (n=5) | 1.75 | 0.21 | 0.46 |
| (n=5) Zm3 - Ec4 (n=5) | 1.65 | 0.21 | 0.52 |
| (n=5) Zm3 - Ec5 (n=5) | 1.50 | 0.21 | 0.53 |
|  |  |  |  |
| (n=5) Zm4 - Ec1 (n=5) | 2.12 | 0.27 | 0.32 |
| (n=5) Zm4 - Ec2 (n=5) | 1.43 | 0.27 | 0.42 |
| (n=5) Zm4 - Ec3 (n=5) | 1.29 | 0.27 | 0.46 |
| (n=5) Zm4 - Ec4 (n=5) | 1.22 | 0.27 | 0.52 |
| (n=5) Zm4 - Ec5 (n=5) | 1.12 | 0.27 | 0.53 |
|  |  |  |  |
| (n=5) Zm5 - Ec1 (n=5) | 1.86 | 0.25 | 0.32 |
| (n=5) Zm5 - Ec2 (n=5) | 1.32 | 0.25 | 0.42 |
| (n=5) Zm5 - Ec3 (n=5) | 1.19 | 0.25 | 0.46 |
| (n=5) Zm5 - Ec4 (n=5) | 1.17 | 0.25 | 0.52 |
| (n=5) Zm5 - Ec5 (n=5) | 1.13 | 0.25 | 0.53 |
|  |  |  |  |
| (n=5) Zm6 - Ec1 (n=5) | 1.29 | 0.31 | 0.32 |
| (n=5) Zm6 - Ec2 (n=5) | 1.06 | 0.31 | 0.42 |
| (n=5) Zm6 - Ec3 (n=5) | 1.00 | 0.31 | 0.46 |
| (n=5) Zm6 - Ec4 (n=5) | 1.04 | 0.31 | 0.52 |
| (n=5) Zm6 - Ec5 (n=5) | 1.07 | 0.31 | 0.53 |
|  |  |  |  |
| (n=5) Zm7 - Ec1 (n=5) | 1.93 | 0.28 | 0.32 |
| (n=5) Zm7 - Ec2 (n=5) | 1.36 | 0.28 | 0.42 |
| (n=5) Zm7 - Ec3 (n=5) | 1.24 | 0.28 | 0.46 |
| (n=5) Zm7 - Ec4 (n=5) | 1.18 | 0.28 | 0.52 |
| (n=5) Zm7 - Ec5 (n=5) | 1.13 | 0.28 | 0.53 |
|  |  |  |  |
|  |  |  |  |
| **maize - b. grass (united exp. 2)** | All | Maize | B. grass |
| (n=5) Zm1 - Ec1 (n=5) | 1.52 | 0.23 | 0.55 |
| (n=5) Zm1 - Ec2 (n=5) | 1.75 | 0.23 | 0.42 |
| (n=5) Zm1 - Ec3 (n=5) | 1.16 | 0.23 | 0.52 |
| (n=5) Zm1 - Ec4 (n=5) | 1.16 | 0.23 | 0.60 |
| (n=5) Zm1 - Ec5 (n=5) | 1.21 | 0.23 | 0.58 |
|  |  |  |  |
| (n=5) Zm2 - Ec1 (n=5) | 1.31 | 0.34 | 0.55 |
| (n=5) Zm2 - Ec2 (n=5) | 1.53 | 0.34 | 0.42 |
| (n=5) Zm2 - Ec3 (n=5) | 0.90 | 0.34 | 0.52 |
| (n=5) Zm2 - Ec4 (n=5) | 0.87 | 0.34 | 0.60 |
| (n=5) Zm2 - Ec5 (n=5) | 0.90 | 0.34 | 0.58 |
|  |  |  |  |
| (n=5) Zm3 - Ec1 (n=5) | 1.56 | 0.29 | 0.55 |
| (n=5) Zm3 - Ec2 (n=5) | 1.77 | 0.29 | 0.42 |
| (n=5) Zm3 - Ec3 (n=5) | 1.20 | 0.29 | 0.52 |
| (n=5) Zm3 - Ec4 (n=5) | 1.20 | 0.29 | 0.60 |
| (n=5) Zm3 - Ec5 (n=5) | 1.24 | 0.29 | 0.58 |
|  |  |  |  |
| (n=5) Zm4 - Ec1 (n=5) | 1.25 | 0.27 | 0.55 |
| (n=5) Zm4 - Ec2 (n=5) | 1.49 | 0.27 | 0.42 |
| (n=5) Zm4 - Ec3 (n=5) | 0.93 | 0.27 | 0.52 |
| (n=5) Zm4 - Ec4 (n=5) | 0.95 | 0.27 | 0.60 |
| (n=5) Zm4 - Ec5 (n=5) | 1.01 | 0.27 | 0.58 |
|  |  |  |  |
| (n=5) Zm5 - Ec1 (n=5) | 1.15 | 0.27 | 0.55 |
| (n=5) Zm5 - Ec2 (n=5) | 1.37 | 0.27 | 0.42 |
| (n=5) Zm5 - Ec3 (n=5) | 0.87 | 0.27 | 0.52 |
| (n=5) Zm5 - Ec4 (n=5) | 0.88 | 0.27 | 0.60 |
| (n=5) Zm5 - Ec5 (n=5) | 0.98 | 0.27 | 0.58 |
|  |  |  |  |
| (n=5) Zm6 - Ec1 (n=5) | 0.95 | 0.30 | 0.55 |
| (n=5) Zm6 - Ec2 (n=5) | 1.25 | 0.30 | 0.42 |
| (n=5) Zm6 - Ec3 (n=5) | 0.80 | 0.30 | 0.52 |
| (n=5) Zm6 - Ec4 (n=5) | 0.84 | 0.30 | 0.60 |
| (n=5) Zm6 - Ec5 (n=5) | 0.94 | 0.30 | 0.58 |
|  |  |  |  |
| (n=5) Zm7 - Ec1 (n=5) | 1.23 | 0.32 | 0.55 |
| (n=5) Zm7 - Ec2 (n=5) | 1.39 | 0.32 | 0.42 |
| (n=5) Zm7 - Ec3 (n=5) | 0.96 | 0.32 | 0.52 |
| (n=5) Zm7 - Ec4 (n=5) | 0.95 | 0.32 | 0.60 |
| (n=5) Zm7 - Ec5 (n=5) | 1.06 | 0.32 | 0.58 |
