# Supplementary figures and images for: Root Differentiation of Agricultural Plant Cultivars and Proveniences Using FTIR Spectroscopy
Source: Front Plant Sci. 2018 Jun 5;9:748. doi: 10.3389/fpls.2018.00748 (PMC6008560; doi:10.3389/fpls.2018.00748)

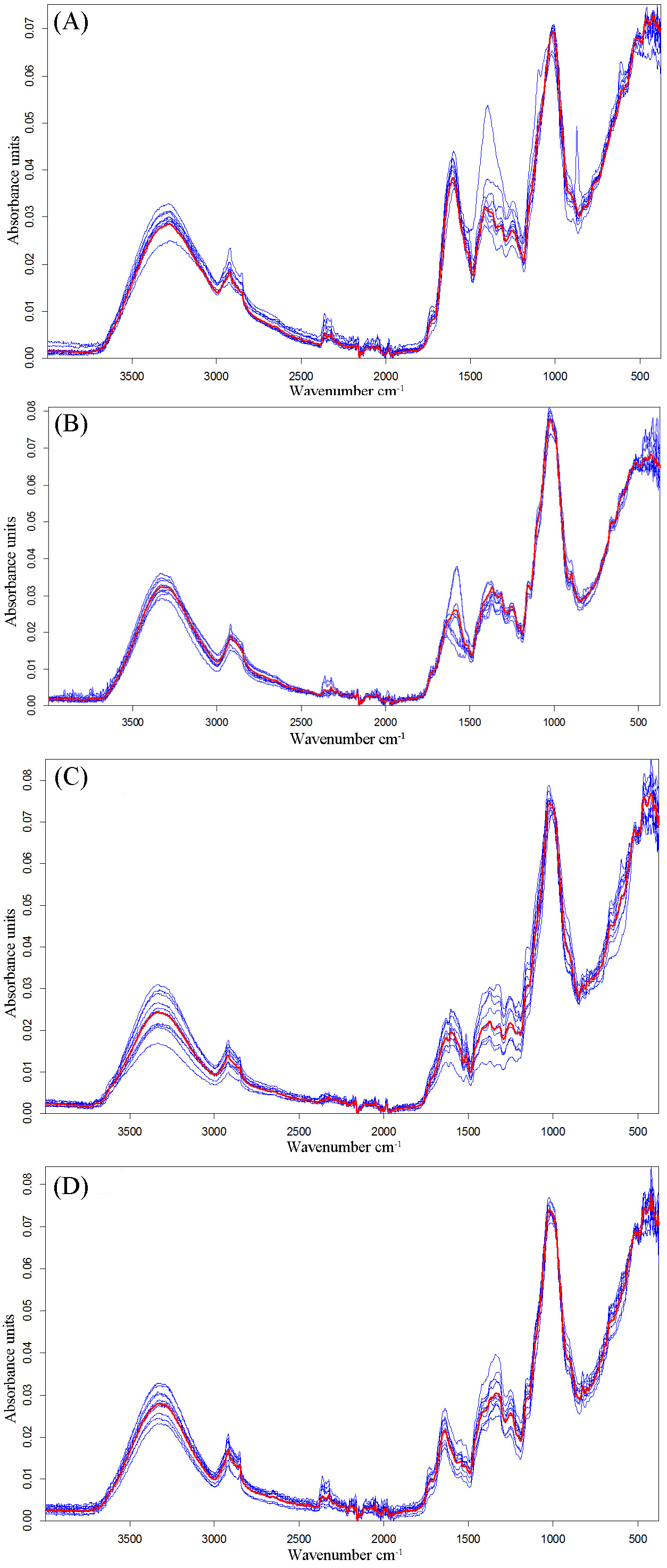

Supplement: Supplementary file 3 [file Image_1.JPEG]

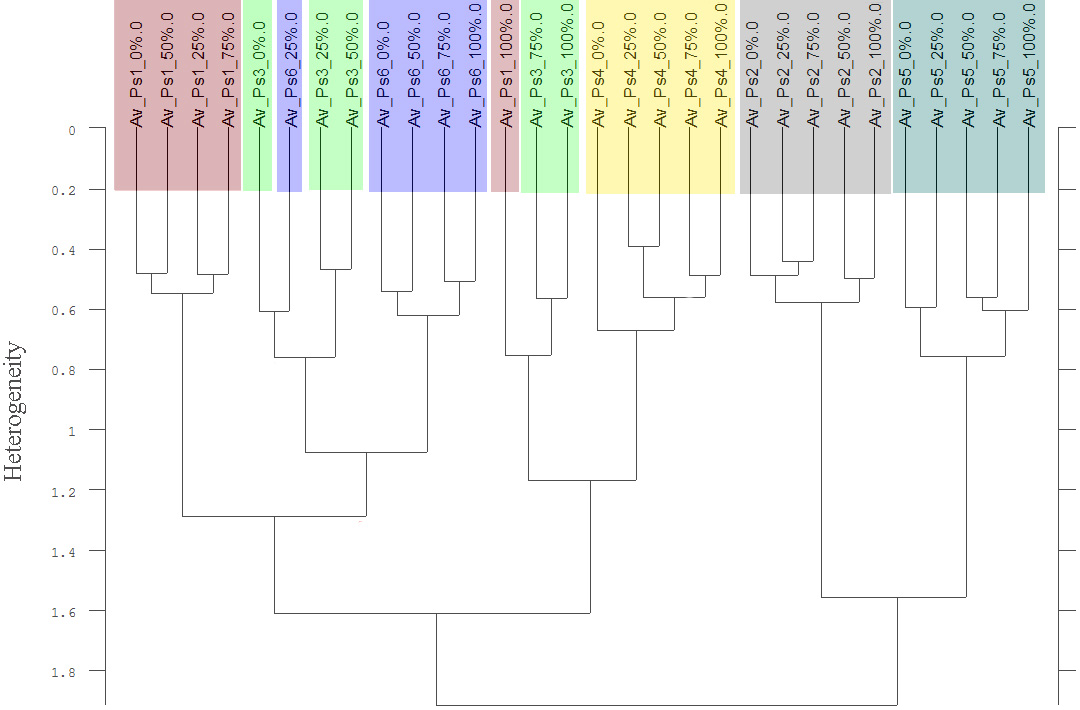

Supplement: Supplementary file 4 [file Image_2.JPEG]
